# Extreme Prematurity and Pulmonary Outcomes Program in Saitama: Protocol for a Prospective Multicenter Cohort Study in Japan

**DOI:** 10.2196/22948

**Published:** 2021-03-05

**Authors:** Fumihiko Namba, Kosuke Tanaka, Sayu Omori, Kazushige Ikeda, Ken Kawabata, Hiroaki Sato, Masakazu Honda, Tomonori Ichikawa, Yoshihiro Minosaki, Takehiro Michikawa, Shuntaro Oka, Kazuhiko Kabe

**Affiliations:** 1 Department of Pediatrics Saitama Medical Center Saitama Medical University Saitama Japan; 2 Division of Neonatology Department of Pediatrics Saitama City Hospital Saitama Japan; 3 Division of Neonatology Saitama Children's Medical Center Saitama Japan; 4 Division of Neonatology Department of Perinatal and Neonatal Medicine Saitama Medical Center, Jichi Medical University Saitama Japan; 5 Division of Neonatal Medicine Department of Pediatrics Saitama Medical University Hospital Saitama Japan; 6 Neonatal Intensive Care Unit Kawaguchi Municipal Medical Center Saitama Japan; 7 Department of Environmental and Occupational Health School of Medicine Toho University Tokyo Japan

**Keywords:** prematurity, preterm infant, bronchopulmonary dysplasia, respiratory outcome

## Abstract

**Background:**

Because of the improvements in survival rates for preterm infants, not only the rates of bronchopulmonary dysplasia (BPD) but also those of long-term respiratory complications of premature birth are increasing, resulting in financial and health burdens in developed countries. Thus far, the risk factors of respiratory morbidities in extremely preterm infants remain unknown. Furthermore, the definition and the predictive ability of BPD for long-term respiratory outcomes are yet to be determined.

**Objective:**

The objective of our study, Extreme Prematurity and Pulmonary Outcomes Program in Saitama, is to develop the diagnostic criteria for BPD and to determine the prognostic factors contributing to the long-term pulmonary outcomes manifesting in extremely preterm infants.

**Methods:**

The Extreme Prematurity and Pulmonary Outcomes Program in Saitama is an observational prospective cohort study performed by a consortium of six neonatal intensive care units (NICUs) in Saitama, Japan. The subjects included in this study are infants (from each clinical center) with gestational ages 22 to 27 weeks. The target is 400 subjects. This study aims to determine the definition of BPD and other perinatal factors that accurately predict the long-term pulmonary outcomes in survivors of extreme prematurity. Moreover, the association between BPD and postprematurity respiratory disease will be investigated using generalized linear models.

**Results:**

The protocol and consent forms were evaluated and approved on September 5, 2019, by the Ethics Committee of Saitama Medical Center, Saitama Medical University. Enrollment began on April 1, 2020. It is expected to end on March 31, 2023. The follow-up for 1 year corrected age is expected to continue through the middle of 2024.

**Conclusions:**

The Extreme Prematurity and Pulmonary Outcomes Program in Saitama incorporates aspects of neonatal care in secondary- and tertiary-level NICUs to develop existing research studies on the definition of BPD, objective biomarkers, and outcome measures of respiratory morbidity in extremely preterm infants beyond NICU hospitalization, thereby leading to a novel understanding of the nature and natural history of BPD and potential mechanistic and therapeutic targets in at-risk subjects.

**International Registered Report Identifier (IRRID):**

DERR1-10.2196/22948

## Introduction

**Figure 1:**
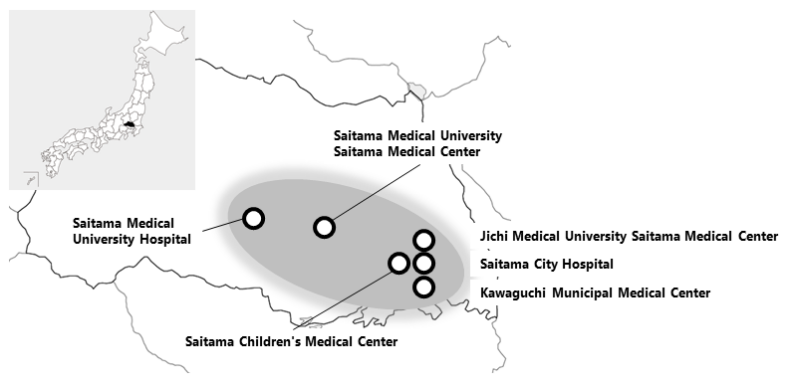
Centers participating in the study. The map has been taken from https://n.freemap.jp/. The copyright belongs to FN.

The rate of preterm birth has been increasing over the past several decades [1]. Preterm birth is associated with serious respiratory diseases, such as bronchopulmonary dysplasia (BPD), which can be problematic during the first 2 years of life and even in the teen and adult years [2]. Defining the diagnostic criteria for BPD and a better understanding of the etiologies and risk factors of respiratory diseases associated with prematurity, especially BPD, are important to effectively prevent and treat long-term pulmonary morbidities [2]. It is well known that the chance of developing BPD in preterm infants is inversely related to their gestational age. However, other reliable clinical markers to estimate the severity of future disease or predict the likelihood of infants developing long-term respiratory complications are insufficient, and objective biochemical or physiologic indexes for clinical or research purposes are also uncommon. Considering the gaps in definitional, operational, and mechanistic understanding, Maitre et al created the Prematurity and Respiratory Outcomes Program (PROP), a prospective multicenter study of respiratory outcomes among preterm infants in the United States, with the goal to determine the characteristics of persistent pulmonary diseases associated with prematurity and develop the means of predicting the risk factors of the disease [3,4]. They concluded that both BPD and perinatal clinical data, including male sex, smoking during pregnancy, infant race, public insurance, birth weight, and parent with asthma, accurately determined extremely preterm infants at risk for persistent and severe respiratory morbidity at 1 year [5]. However, the definition and predictive ability of BPD regarding long-term respiratory outcomes are yet to be determined [6,7]. Therefore, we created the Extreme Prematurity and Pulmonary Outcomes Program in Saitama to determine the diagnostic criteria for BPD and the prognostic factors contributing to the 1-year respiratory morbidity in a cohort of more than 400 extremely preterm infants. The Extreme Prematurity and Pulmonary Outcomes Program in Saitama will be implemented in six neonatal intensive care units (NICUs), including two tertiary-level and four secondary-level NICUs, located in Saitama, Japan (Figure 1). The data collected from the Extreme Prematurity and Pulmonary Outcomes Program in Saitama will also be used to produce a high-resolution phenotype of the disease, which can be used clinically, especially in future clinical trials.

The study protocol shows the design of the Extreme Prematurity and Pulmonary Outcomes Program in Saitama study and the breadth of data that will be obtained throughout the NICU stay and 1-year follow-up period. In this study, we aim to develop the diagnostic criteria for BPD and to determine the prognostic factors contributing to the long-term pulmonary outcomes manifesting in extremely preterm infants.

## Methods

### Participating Centers and Their Roles and Responsibilities

The Extreme Prematurity and Pulmonary Outcomes Program in Saitama required each participating center to follow a multisite shared protocol for measuring the respiratory phenotypes and outcomes of extremely preterm infants. A total of five NICUs (three secondary-level and two tertiary-level NICUs) monitor the activities of all extremely preterm infants (<28 weeks) born in Saitama, Japan (Figure 1), and each NICU contributes to the data collection, coordination, and oversight of the multicenter components. Saitama Medical Center, Saitama Medical University manages the clinical report forms, provides support for the standardization of definitions and data collection, identifies and resolves issues, encourages the other participating NICUs to report updates of the project, and determines the necessary steps to be taken by the NICUs. The Extreme Prematurity and Pulmonary Outcomes Program in Saitama working group developed the initial protocols for maternal and neonatal database elements (Multimedia Appendix 1, Multimedia Appendix 2, Multimedia Appendix 3, Multimedia Appendix 4, and Multimedia Appendix 5) and consulted about the protocol of the PROP study regarding respiratory measurements, such as the oxygen requirement challenge test [4].

### Multicenter Protocol Development

#### Primary and Secondary Outcomes

The aims of the Extreme Prematurity and Pulmonary Outcomes Program in Saitama are to determine the definition of BPD and to identify early clinical, physiological, or biochemical biomarkers during the NICU hospitalization, which are deemed to predict respiratory morbidity throughout the first year of life. The primary outcome observed by the Extreme Prematurity and Pulmonary Outcomes Program in Saitama is the presence or absence of postprematurity respiratory disease (PRD), a composite outcome that will be obtained from longitudinal data in the first year after discharge from the NICU [4]. Morbidity in the following four domains will be examined: (1) respiratory symptoms; (2) medication use; (3) hospitalizations; and (4) dependence on technology during the first year of life. Mortality from a cardiorespiratory cause will be considered as well.

Secondary outcomes include respiratory morbidity severity [5], premature infant respiratory status, chronic respiratory morbidity (CRM) 1, CRM 2, death, neurodevelopmental outcomes, visual and hearing impairments, time to final weaning off from oxygen therapy, and time to final weaning off from respiratory technology support.

### Protocol

**Figure 2:**
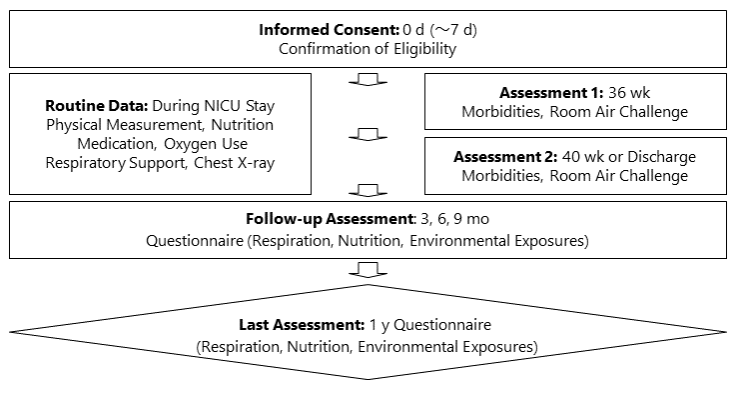
Protocol outline. NICU: neonatal intensive care unit.

The inclusion and exclusion criteria are listed in Textbox 1, and the protocol is outlined in Figure 2. The sample size for this multicenter cohort study is prespecified to include 400 extremely preterm infants at the birth time point (280 [70%] infants with BPD and 120 [30%] infants without BPD, based on our previous study [8]). The difference in PRD occurrence between BPD and non-BPD patients will be compared. This sample size has a statistical power of 80% to detect a difference in PRD occurrence between BPD and non-BPD patients (PRD occurrence is roughly 80% in BPD patients and 60% in non-BPD patients, based on the PROP study [5]).

**Inclusion and exclusion criteria.**

**Inclusion criteria**

- Informed consent obtained and consent form signed
- 7 days of age or less at enrollment
- 22 0/7 to 27 6/7 weeks using the best obstetrical estimate

**Exclusion criteria**

- Structurally significant congenital heart disease
- Structural abnormalities of the upper airway, lung, or chest wall
- Congenital malformation or syndrome that adversely affects life expectancy or cardiopulmonary development
- Family unlikely to be available for long-term follow-up
- Considered by the investigator to be an unsuitable candidate for this study

### Clinical Data Collection, Management, and Storage Systems

All data are prospectively collected from birth through medical record reviews and family interviews, and include maternal and infant demographics; clinical data and comorbidities; and daily infant respiratory, nutritional, and medication data throughout the NICU stay (Multimedia Appendix 1, Multimedia Appendix 2, Multimedia Appendix 3, Multimedia Appendix 4, and Multimedia Appendix 5). After discharge from the NICU, a series of questionnaires, in accordance with that of the PROP study [4], is used. The questionnaires assess the domains of respiratory morbidity at 3, 6, 9, and 12 months corrected age. At 6 and 12 months, a survey on environmental respiratory irritant exposures, including tobacco smoke and animal substances, is conducted as well. An in-person visit for physical examination and history taking is performed at 1 year corrected age.

NICUs contribute and retain access to their own data through an electronic data capture software (NICU Data Management System [NDMS], Education Software Co, Ltd). In each NICU, all the data, without identifying personal information, are exported to a CSV file, and the file is sent to Saitama Medical Center, Saitama Medical University.

### Assessments of Physiological Room-Air Challenge

To physiologically define and classify BPD, data regarding the requirement of supplemental oxygen estimated by the standardized oxygen requirement challenge test at 36 weeks postmenstrual age (PMA) and at 40 weeks PMA or discharge (whichever comes first) are collected. The protocol developed by Walsh et al [9] was modified to effectively discriminate between the effects of FiO_2_ and flow [4] (Multimedia Appendix 6).

### Analytical Approaches

The subjects are classified into the following two groups: BPD and non-BPD. Concerning the maternal and neonatal background information, the frequency and percentage in each group are calculated for discrete data, whereas descriptive statistic values in each group, such as mean and SD, and median and IQR, are calculated for continuous data.

The primary outcome of PRD requires that infants demonstrate pulmonary morbidity in one of the following four domains that are examined after discharge from the NICU: (1) respiratory medications; (2) hospitalization from a cardiopulmonary cause; (3) coughing, wheezing, or other respiratory symptoms; and (4) home technology dependence in at least two time frames (at 3-month intervals) during the first year of life. Death from a respiratory cause is also included. We will apply generalized linear models to treat repeated measures and check the statistical difference in the PRD incidence in the presence and absence of BPD after adjustment for several covariates. Gestational age and variables with a P value <.05 in univariate analyses will be considered for inclusion in multivariate models. Missing values are not complemented, because the number of cases of missing values is extremely small. When the outliers other than abnormal values with apparent causes are excluded, their handling procedure is determined by blind review of the data, and the statistical analysis plan is revised.

To analyze the potential influence of gestational age on respiratory outcomes, a subgroup analysis will be conducted. Based on the gestational age at birth, the subjects will be divided into the following three subgroups: (1) 22 and 23 weeks of gestation, (2) 24 and 25 weeks of gestation, and (3) 26 and 27 weeks of gestation.

### Study Approval by Institutional Review Boards

The multicenter Extreme Prematurity and Pulmonary Outcomes Program in Saitama protocol and consent forms were evaluated by the Ethics Committee of Saitama Medical Center, Saitama Medical University (initial approval date: September 5, 2019). In addition, the institutional review board at each Extreme Prematurity and Pulmonary Outcomes Program in Saitama site determined the risk level, based on local interpretation. The complete list of institutional review board approvals and protocol numbers can be found in Multimedia Appendix 7.

### Patient and Public Involvement

Patients or the public were not involved in the design, conduct, reporting, or dissemination plans of our research.

## Results

Written informed consent will be obtained from the parents of the infants enrolled in this study. Enrollment began on April 1, 2020, in Saitama Medical Center, Saitama Medical University, and May 1, 2020, in the other NICUs. It is expected to end on March 31, 2023. The follow-up for 1 year corrected age is expected to continue through the middle of 2024. Four and eight eligible extremely preterm infants were enrolled in April and May 2020, respectively.

## Discussion

We are conducting a prospective multicenter cohort study to develop the diagnostic criteria for BPD and to determine the prognostic factors contributing to the long-term pulmonary outcomes manifesting in extremely preterm infants. The definition of BPD has gradually evolved, since the initial description of the disease in 1967 by Northway et al [10]. Shennan et al suggested the definition of supplemental oxygen requirements at 36 weeks PMA [11]. In 2000, the severity-based definition, which categorizes BPD as mild, moderate, or severe according to the respiratory support provided at 36 weeks PMA among very preterm infants treated with supplemental oxygen for at least 28 days, was constructed (NIH consensus definition) [12]. However, the validity and utility of these commonly used definitions have been questioned, because it has been reported that (1) there is an inconsistent correlation with long-term respiratory outcomes [13], (2) oxygen/respiratory support at 40 weeks, not at 36 weeks, is the best predictor for serious respiratory morbidity [7], and (3) regardless of supplemental oxygen use, respiratory support at 36 weeks PMA best predicted early childhood morbidity [6]. Therefore, the Extreme Prematurity and Pulmonary Outcomes Program in Saitama, a prospective multicenter cohort study of extremely preterm infants in Japan, will be conducted in order to provide a novel definition of BPD that can best predict the long-term pulmonary outcomes throughout the first year of life and a better understanding of the mechanisms, evolution, and consequences of lung diseases among extremely preterm infants.

## Appendix

Multimedia Appendix 1 Extreme Prematurity and Pulmonary Outcomes Program in Saitama: baby’s baseline data.

Multimedia Appendix 2 Extreme Prematurity and Pulmonary Outcomes Program in Saitama: daily growth and nutrition/daily medication data.

Multimedia Appendix 3 Extreme Prematurity and Pulmonary Outcomes Program in Saitama: comorbidities of prematurity.

Multimedia Appendix 4 Extreme Prematurity and Pulmonary Outcomes Program in Saitama: standard Visit.

Multimedia Appendix 5 Extreme Prematurity and Pulmonary Outcomes Program in Saitama: brain imaging data.

Multimedia Appendix 6 Oxygen requirement challenge test.

Multimedia Appendix 7 Institutional review board protocols at individual Extreme Prematurity and Pulmonary Outcomes Program in Saitama sites.

## Abbreviations

BPD: bronchopulmonary dysplasia
CRM: chronic respiratory morbidity
NICU: neonatal intensive care unit
PMA: postmenstrual age
PRD: postprematurity respiratory disease
PROP: Prematurity and Respiratory Outcomes Program

## References

[1] Sakata S, Konishi S, Ng CFS, Watanabe C (2018). Preterm birth rates in Japan from 1979 to 2014: Analysis of national vital statistics. J Obstet Gynaecol Res.

[2] Hilgendorff A, O'Reilly MA (2015). Bronchopulmonary dysplasia early changes leading to long-term consequences. Front Med (Lausanne).

[3] Maitre NL, Ballard RA, Ellenberg JH, Davis SD, Greenberg JM, Hamvas A, Pryhuber GS, PrematurityRespiratory Outcomes Program (2015). Respiratory consequences of prematurity: evolution of a diagnosis and development of a comprehensive approach. J Perinatol.

[4] Pryhuber GS, Maitre NL, Ballard RA, Cifelli D, Davis SD, Ellenberg JH, Greenberg JM, Kemp J, Mariani TJ, Panitch H, Ren C, Shaw P, Taussig LM, Hamvas A, PrematurityRespiratory Outcomes Program Investigators (2015). Prematurity and respiratory outcomes program (PROP): study protocol of a prospective multicenter study of respiratory outcomes of preterm infants in the United States. BMC Pediatr.

[5] Keller RL, Feng R, DeMauro SB, Ferkol T, Hardie W, Rogers EE, Stevens TP, Voynow JA, Bellamy SL, Shaw PA, Moore PE, PrematurityRespiratory Outcomes Program (2017). Bronchopulmonary Dysplasia and Perinatal Characteristics Predict 1-Year Respiratory Outcomes in Newborns Born at Extremely Low Gestational Age: A Prospective Cohort Study. J Pediatr.

[6] Jensen EA, Dysart K, Gantz MG, McDonald S, Bamat NA, Keszler M, Kirpalani H, Laughon MM, Poindexter BB, Duncan AF, Yoder BA, Eichenwald EC, DeMauro SB (2019). The Diagnosis of Bronchopulmonary Dysplasia in Very Preterm Infants. An Evidence-based Approach. Am J Respir Crit Care Med.

[7] Isayama T, Lee SK, Yang J, Lee D, Daspal S, Dunn M, Shah PS, Canadian Neonatal NetworkCanadian Neonatal Follow-Up Network Investigators (2017). Revisiting the Definition of Bronchopulmonary Dysplasia: Effect of Changing Panoply of Respiratory Support for Preterm Neonates. JAMA Pediatr.

[8] Miyake F, Ito M, Minami H, Tamura M, Namba F (2020). Management of bronchopulmonary dysplasia in Japan: A 10-year nationwide survey. Pediatr Neonatol.

[9] Walsh MC, Wilson-Costello D, Zadell A, Newman N, Fanaroff A (2003). Safety, reliability, and validity of a physiologic definition of bronchopulmonary dysplasia. J Perinatol.

[10] Northway WH, Rosan RC, Porter DY (1967). Pulmonary disease following respirator therapy of hyaline-membrane disease. Bronchopulmonary dysplasia. N Engl J Med.

[11] Shennan AT, Dunn MS, Ohlsson A, Lennox K, Hoskins EM (1988). Abnormal pulmonary outcomes in premature infants: prediction from oxygen requirement in the neonatal period. Pediatrics.

[12] Jobe AH, Bancalari E (2001). Bronchopulmonary dysplasia. Am J Respir Crit Care Med.

[13] Lefkowitz W, Rosenberg SH (2008). Bronchopulmonary dysplasia: pathway from disease to long-term outcome. J Perinatol.

