# Supplementary material for: Extreme Prematurity and Pulmonary Outcomes Program in Saitama: Protocol for a Prospective Multicenter Cohort Study in Japan
Source: JMIR Res Protoc. 2021 Mar 5;10(3):e22948. doi: 10.2196/22948 (PMC7980118; doi:10.2196/22948)
Supplement: Multimedia Appendix 1 [file resprot_v10i3e22948_app1.docx]

EXTREME PREMATURITY AND PULMONARY OUTCOMES PROGRAM IN SAITAMA

BABY’S BASELINE DATA

PID: _______________ DATE: _____/______/_______

1. What is the baby’s gender? □Male, □Female, □Unknown
2. What is the baby’s birth weight? __________ g
3. What is the baby’s birth height? __________ cm
4. What is the baby’s head circumference? __________ cm
5. What was the baby’s birth location?

□ Born inside the study center, □ Born outside the study center

1. What was the baby’s expected date of birth (EDC)? ____/_____/_____
2. Was this a multiple birth? □Yes, □No

If Yes, answer questions 7a and 7b.

7a. Indicate the baby’s birth order: __ Number of __ Number

7b. Record the PID(s) of siblings enrolled in the EPOPS: PID__________

1. Were umbilical artery blood gas analyses performed? □Yes, □No

If Yes, record the result(s) of the umbilical artery blood gas analysis:

8a. Sample pH: __._____

8b. Sample Base Deficit: _____.__

1. What was the Apgar Score?

9a. 1min: __

9b. 5min: __

9c. 10min: __

1. 10a. Was umbilical cord milking performed? □Yes, □No

10b. Was delayed umbilical cord clamping performed? □Yes, □No

1. Were any stabilization procedures provided at birth? □Yes, □No

11a. If Yes, check all that apply:

□ Supplemental O2

□ CPAP

□ Non-invasive positive pressure ventilation with flow inflating or self inflating bag

□ T-Piece resuscitator

□ Intubation

□ Chest compression

□ Cardiac drugs (Epinephrine)

□ Surfactant administration

□ INSURE method (Intubation, Surfactant administration and extubation)

1. Who performed resuscitation? ___

□ neonatal specialist, □ pediatric specialist, □ trainee, □ others

1. What was the first temperature recorded at the first NICU admission?

Core temperature (e.g. rectal): ____.__℃

Peripheral / skin temperature (e.g. axillary): ____.__℃

1. What was the first blood glucose level recorded at the first NICU admission? _______mg/dl

14a. Indicate how the blood glucose level was obtained:

□ venous, □ arterial, □ heel-cut

1. What was the first blood pressure recorded at the first NICU admission? ____/____mmHg

15a. Indicate how the blood pressure was obtained:

□ invasive(arterial), □ noninvasive,

1. Was Prophylactic Indomethacin given within the first 24-hours of life? □Yes, □No

EXTREMELY PREMATURITY AND PULMONARY OUTOCOMES PROGRAM IN SAITAMA

SCREENING FOR ELIGIBILITY AND CONSENT

PID: _______________ DATE: _____/______/_______

1. What is the name of your facility? __________
2. What is the baby’s date of birth? ____/_____/_____
3. What is the baby’s time of birth? ___:___
4. Fathers Ethnicity

□ Asian, □ Black / African, □ White / Caucasian, □ Others

1. Mothers Ethmicity

□ Asian, □ Black / African, □ White / Caucasian, □ Others

Inclusion Criteria

1. Is the baby’s Gestational Age (GA) between 22 weeks and 0/7 days and 27 weeks and 6/7 days?

7a. Indicate the number of completed weeks and the number of completed days: ___Weeks__Day

7b. What method was used to determine the Gestational Age of the baby?

□ Early dating ultrasound (<20 weeks)

□ Certain LMP date

□ Best clinical estimate

1. Is the baby’s postnatal age less than 7 Days? □Yes, □No

Exclusion Criteria

1. Is the baby considered not to be viable (decision not to administer effective therapies)?

□Yes, □No

1. Does the baby have congenital heart disease (not including PDA and hemodynamically insignificant VSD or ASD)? □Yes, □No
2. Does the baby have any structural abnormalities of the upper airway or lungs?

□Yes, □No

1. Does the baby have any other congenital malformations or syndromes that adversely affect life expectancy or development? □Yes, □No
2. Is the baby unlikely to be available for long-term follow-up? □Yes, □No

Eligibility

1. Does the baby meet both of the inclusion criteria and none of the exclusion criteria and is therefore eligible for the Database?

Complete the Consent Section only for babies who are eligible for the EPOP Database.

1. Were the parents of the baby approached about the study?

14a. If No, select the primary response that best explains why the parents were not approached:

□ Research staff was not available

□ Parents were not available

□ Screening oversight

□ On request of responsible physician

□ Other, specify:_________________

14b. If Yes, was parental consent obtained? □Yes, □No

14c. If Question 14 b is No, select the primary response that best explains why consent was not obtained:

□ Parents object to participation in research studies

□ Baby was enrolled in another research study

□ Parents objected to long term follow-up

□ Other, specify:_________________

Enrollment

1. Was the baby enrolled into the study? □Yes, □No

15a. If Yes, enter Date of Enrollment: ____/_____/_____

EXTREMELY PREMATURITY AND PULMONARY OUTOCOMES PROGRAM IN SAITAMA

MATERNAL BASELINE DATA

PID: _______________ DATE: _____/______/_______

Maternal Demographic Data:

1. What is the mother’s date of birth? _____/______/_______
2. What is the mother’s pre-pregnancy weight? ____._kg
3. What is the mother’s height? _____._cm
4. What is the family arrangement?

□ Single parent family, □ Two parent family, □ Unknown

Pregnancy History

1. Is this pregnancy after fertility treatment? □Yes, □No

5a. If Yes, what treatment did you have.

□ Timed Intercourse

□ Stimulating ovulation with fertility drugs

□ Intrauterine insemination (IUI)

□ In vitro fertilization (IVF)

□ Others

1. Did the mother have diabetes during pregnancy? □Yes, □No

6a. If Yes, did she receive insulin for her diabetes? □Yes, □No

1. Did the mother have hypertension during pregnancy? □Yes, □No

7a. If Yes, did she receive medication to treat her hypertension? □Yes, □No

1. Did the mother have asthma during pregnancy? □Yes, □No

8a.If Yes, did she take medication regularly to control her asthma? □Yes, □No

1. Did the mother have any infections in pregnancy? □Yes, □No

9a. If Yes, check all that apply:

□ Chlamydia □ Toxoplasmosis □ Rubella □ Hepatitis B

□ Hepatitis C □ Genital herpes □ Cytomegalovirus (CMV) □ HIV

□ Chickenpox □ HTLV-1 □ Syphilis □ Parvovirus B19

1. Did the mother take any medications to prolong pregnancy?

10a. If Yes, check all that apply:

□ Progesterone

□ Cyclooxygenase inhibitors (Indomethacin)

□ Intravenous infusion of betamimetics

□ Oral betamimetics

□ Calcium Channel Blockers

□ Oxytocin receptor antagonist

□ Magnesium Sulfate

□ Other:______________

1. Dose the mother smoke tobacco products?

□ Yes, □ Quit smoking during pregnancy, □ No, but had smoking habit in the past, □ Never

11a. Did anyone else smoke tobacco regularly in the mother’s home during her pregnancy?

□Yes, □No

1. Dose the mother drink alcohol?

□ Yes, □ Quit drinking during pregnancy, □ No, but had drinking habit in the past, □ Never

1. Indicate the mother’s height and weight at the time of delivery

Height: _____.__cm □ Unknown

Weight: ___._ kg □ Unknown

Labor and Delivery Data:

1. Was there placental abruption? □Yes, □No, □Unknown
2. Did the membrane rupture > 18 hours before delivery? □Yes, □No, □Unknown

15a. If Question 18 is Yes, did the membrane rupture more than 7 days before delivery?

□Yes, □No, □Unknown

1. Was there any clinical chorioamnionitis? □Yes, □No, □Unknown

16a. Was there fever over 38 degrees? □Yes, □No, □Unknown

16b. Was there uterine contraction difficult to suppress? □Yes, □No, □Unknown

16c. Was there maternal tachycardia? □Yes, □No, □Unknown

16d. Was maternal white blood cell count >15000/ml? □Yes, □No, □Unknown

16e. Was vaginal discharge foul-smelling? □Yes, □No, □Unknown

16f. If Yes, was placental pathology obtained? □Yes, □No, □Unknown

16g. If Question 16f is Yes, was there histologic evidence of chorioamnionitis?

□Yes, □No, □Unknown

16h. If Question 16g is Yes, what was the grade of the chorioamnionitis in Blanc's classification?

16i. If Question 16f is Yes, was there histologic evidence of funisitis?

1. Were antibiotics given? □Yes, □No, □Unknown

17a. If Yes, why were antibiotics given (check all that apply)?

□ Chorioamnionitis

□ Group B Streptococcus (GBS) prophylaxis

□ Preterm labor

□ Other:___________

1. Were antenatal corticosteroids given? □Yes, □No, □Unknown

18a. If Yes, total number of completed courses:

□ None, □ One course, □ Two courses, □ Three courses

□ Four courses, □ Five courses

19a. Number of incomplete courses:

□ None, □ One or more courses

1. Was magnesium sulfate given for any reasons other than tocolysis? □Yes, □No, □Unknown

If Yes, check the primary indication:

□ Preeclampsia / eclampsia, □ Prevention of Cerebral Palsy

1. Was the onset of labor spontaneous? □Yes, □No
2. What was the mode of delivery?

□ Vaginal Vertex, □ Vaginal Breech, □ Caesarian Section
