# Supplementary material for: Extreme Prematurity and Pulmonary Outcomes Program in Saitama: Protocol for a Prospective Multicenter Cohort Study in Japan
Source: JMIR Res Protoc. 2021 Mar 5;10(3):e22948. doi: 10.2196/22948 (PMC7980118; doi:10.2196/22948)
Supplement: Multimedia Appendix 2 [file resprot_v10i3e22948_app2.docx]

EXTREME PREMATURITY AND PULMONARY OUTCOMES PROGRAM IN SAITAMA

DAILY GROWTH AND NUTRITION/DAILY MEDICATION DATA

PID: _______________ DATE: _____/______/_______

Week____ | Day___

Daily Growth and Nutrition Data

1. What was the baby's body weight? ________ g, □ Not done
2. What was the baby's head circumference? ____.__ cm, □ Not done
3. How much milk did the baby receive today?

□ None, □ <120ml/kg/day, □ ≥120ml/kg/day

3a. If any milk feed, indicate the type of milk provided:

□ Human milk, □ Formula, □ Both

3b. If any milk feed, indicate the way of feeding:

□ gastric tube, □ duodenal tube, □ bottle-feeding, □ others

3b. If any milk feed, was thickener used?

□Yes, □No

Daily Medication Data

1. Were any drugs given today? □Yes, □No

If Yes, indicate which of the following drugs were given today:

1. Methylxanthine drugs: □Yes, □No

If Yes, choose one:

□ Caffeine Citrate Dose: _____ mg Given every __ __ hour(s)

Route: □ PO (oral) , □ IV (intravenous)

Loading dose today: □Yes, □No

□ Aminophylline Dose: _____ mg Given every __ __ hour(s)

□ Theophylline Dose: _____ mg Given every __ __ hour(s)

□ Other __________

1. Systemic Corticosteroid drugs: □Yes, □No

If Yes, check all that apply:

□ Hydrocortisone Dose: _____ mg Given every __ __ hour(s)

Route: □ PO (oral) , □ IV (intravenous)

□ Dexamethasone Dose: _____ mg Given every __ __ hour(s)

Route: □ PO (oral) , □ IV (intravenous)

□ Prednisone / Prednisolone Dose: _____ mg Given every __ __ hour(s)

Route: □ PO (oral) , □ IV (intravenous)

□ Methylprednisolone Dose: _____ mg Given every __ __ hour(s)

Route: □ PO (oral) , □ IV (intravenous)

□ Betamethasone Dose: _____ mg Given every __ __ hour(s)

Route: □ PO (oral) , □ IV (intravenous)

□ Other __________

1. Inhaled Steroid drugs: □Yes, □No

7a. If Yes, check all that apply:

□ Budesonide Dose: _____ □ mg □ mcg Given every __ __ hour(s)

□ Fluticasone Dose: _____ □ mg □ mcg Given every __ __ hour(s)

□ Betamethasone Dose: _____ □ mg □ mcg Given every __ __ hour(s)

□ Other __________

1. Inhaled Bronchodilator drugs: □Yes, □No

8a. If Yes, check all that apply:

□ Procaterol Dose: _____ □ mg □ mcg Given every __ __ hour(s)

□ Bromhexine Dose: _____ □ mg □ mcg Given every __ __ hour(s)

□ Other __________

1. Diuretic drugs: □Yes, □No

9a. If Yes, check all that apply:

□ Furosemide Dose: _____ mg Given every __ __ hour(s)

Route: □ PO (oral) , □ IV (intravenous)

□ Spironolactone Dose: _____ mg Given every __ __ hour(s)

Route: □ PO (oral) , □ IV (intravenous)

□ Mannitol Dose: _____ mg Given every __ __ hour(s)

Route: □ PO (oral) , □ IV (intravenous)

□ Canrenoate potassium Dose: _____ mg Given every __ __ hour(s)

Route: □ PO (oral) , □ IV (intravenous)

□ Trichlormethiazide Dose: _____ mg Given every __ __ hour(s)

Route: □ PO (oral) , □ IV (intravenous)

□ Tolvaptan Dose: _____ mg Given every __ __ hour(s)

Route: □ PO (oral) , □ IV (intravenous)

□ Other __________

1. Cardiovascular drugs: □Yes, □No

10a. If Yes, check all that apply:

□ Epinephrine Infusion, □ Dopamine Infusion, □ Dobutamine Infusion

□ Vasopressin, □ Norepinephrine

1. Other Cardio/Respiratory drugs: □Yes, □No

11a. If Yes, check all that apply:

□ Other pulmonary vasodilators for treatment of pulmonary hypertension other than Nitric Oxide

□ Surfactant □ Indomethacin □ Acetaminophen □ Ibuprophen □ Mucolytic

□ Sildenafil □ Milrinone □ Tadalafil □ Bosentan □ Alprostadil

□ Carperitide □ Propranolol □ Nitroglycerin

□ Others ________________________________________________

1. Neuro-Muscular Blocking Agent: □Yes, □No
2. Antimicrobial drugs and other agents to prevent infections: □Yes, □No

13a. If Yes, check all that apply:

□ Antibacterial

□ Ampicillin □Cefotaxime □Cefmetazole □ Arbekacin □‎ Vancomycin

□ Meropenem □‎Cefazolin □ Amikacin □ Gentamicin □ Teicoplanin

□ Sulbactam / Ampicillin □Erythromycin
 □ Others _____________________

□ Antifungal

□ Amphotericin B □ Miconazole □ Fluconazole □ Micafungin □‎ Fosfluconazole

□ Others _____________________

□ Antiviral

□ Aciclovir □Valganciclovir □Ganciclovir □‎Foscarnet

□ Others _____________________

□ Palivizumab

□ Probiotic

1. Anxiolytic, Anticonvulsant, and Narcotic Analgesic drugs: □Yes, □No

14a. If Yes, check all that apply:

□ ‎Diazepam □ Midazolam □ Phenobarbital □ Phenytoin □ Levetiracetam

□ Morphine □ Fentanyl □ Dexmedetomidine □ Triclofos

□Thiopental □ ‎Acetaminophen □ Others _____________________

1. Anti-Gastroesophageal Reflux drugs:

□ Proton Pump Inhibitors (□ Omeprazole, □ Lansoprazole, □ Others ___________)

□ H2 Receptor Antagonists (□ Famotidine, □ Others ___________)

□ Motility agents (□ Mosapride, □ Others ___________)

□ Kampo (□Rikkunshito, □Daikenchuto, □ Others ___________)

□ Others ___________

1. Use of blood products and Hematologic Supplements: □Yes, □No

16a. If Yes, check all that apply:

□ Erythropoietin □ Red Blood Cell □ Transfusion Platelets □ Iron Supplements

□ Others_____________

1. Oral Vitamins and Electrolyte Supplements: □Yes, □No

□ Vitamin D □ Vitamin E □ Multivitamin □ Potassium Supplement

□ Sodium Supplement □ Calcium Supplement □ Phosphate Supplement

□ Magnesium Supplement □ Trace element □ Others_____________

EXTREMELY PREMATURITY AND PULMONARY OUTOCOMES PROGRAM IN SAITAMA

DAILY RESPIRATORY DATA

PID: _______________ DATE: _____/______/_______

Week____ | Day___

1. Did the baby receive any supplemental oxygen today? □Yes, □No

1a. If Yes, how long was supplemental oxygen used for?

□ 12 hours or less □ More than 12 hours

1b. What was the concentration of supplemental oxygen at 1200 (Noon) today?

________%

1. Did the baby receive any other respiratory support today? □Yes, □No

If Questions 1 or 2 are Yes, answer questions 3-6.

1. Was Positive Airway Pressure with Endotracheal Tube used today? □Yes, □No

3a. If Yes, select ventilation mode and record the associated values at 1200 (Noon)

or closest recorded data to 12 (Noon) if noon data are not available.

□ Conventional Mechanical Ventilation (CMV):

Mean Airway Pressure (MAP): _____ .__ cmH2O

Positive End Expiratory Pressure (PEEP): _____ .__ cmH2O

□ High Frequency Oscillation: MAP: _____ .__ cmH2O

□ High Frequency Jet Ventilation: MAP: _____ .__ cmH2O

1. Was Respiratory support without Endotracheal Tube used today? □Yes, □No

4a. If Yes, select ventilation mode and record the associated values at 1200 (Noon)

or closest recorded data to 12 (Noon) if noon data are not available.

□ Nasal Intermittent Mandatory Ventilation (NIMV):

MAP: __ __ . __cmH2O

PEEP: __ __ cmH2O

□ Continuous Positive Airway Pressure (CPAP): CPAP: __ __ cmH2O

□ High Flow Nasal Cannula: Nasal Cannula Flow: __ __ . __ __ __ __ Lpm

□ Nasal Cannula with flow rate: Nasal Cannula Flow: __ __ . __ __ __ __ Lpm

1. Was inhaled nitric oxide given today? □Yes, □No

5a. If Yes, record the concentration at 1200 (Noon) or closest recorded data to

12 (Noon) if noon data are not available.

_______ppm

1. Was the baby reintubated today? □Yes, □No

6a. If Yes, indicate the primary reason why the baby was reintubated:

□ Increasing respiratory distress

□ Stridor

□ Apnea and Bradycardia

□ Suspected infection

□ For diagnostic or therapeutic procedures, including surgery

□ Unplanned extubation(s), indicate the number of occurrences this day: __

Other, specify: ________________________

1. Did the baby have chest X-ray today? □Yes, □No

6a. If Yes, was there diffuse bubbly appearance or irregular funicular emphysematous appearance?

□Yes, □No
