# Supplementary material for: Extreme Prematurity and Pulmonary Outcomes Program in Saitama: Protocol for a Prospective Multicenter Cohort Study in Japan
Source: JMIR Res Protoc. 2021 Mar 5;10(3):e22948. doi: 10.2196/22948 (PMC7980118; doi:10.2196/22948)
Supplement: Multimedia Appendix 3 [file resprot_v10i3e22948_app3.docx]

EXTREME PREMATURITY AND PULMONARY OUTCOMES PROGRAM IN SAITAMA

COMORBIDITIES OF PREMATURITY

PID: _______________ DATE: _____/______/_______

AT WEEK 36, WEEK 40 PMA OR DISCHARGE, WHICHEVER OCCURS FIRST

Cardio-Pulmonary

1. Did the baby have any of the following types of air leaks? □Yes, □No

If Yes, indicate the type(s) of air leak by answering Questions 1a-1d:

1a. Pneumothorax: □Yes, □No

　If Yes, complete 1a1 and 1a2

1a1. Was a chest tube placed? □Yes, □No

1b. Pulmonary Interstitial Emphysema (PIE): □Yes, □No

1c. Pneumomediastinum: □Yes, □No

1d. Pneumopericardium: □Yes, □No

1. Did the baby have any pulmonary hemorrhages? □Yes, □No

If Yes, complete 2a and 2b.

2a. Did these hemorrhages require transfusion of blood products? □Yes, □No

2b. Did these hemorrhages require increased concentrations of supplemental oxygen and / or ventilator support? □Yes, □No

1. Did the baby have Patent Ductus Arteriosus (PDA) requiring any treatment? □Yes, □No

Indicate if any of the following treatments were used to treat the PDA:

□ indomethacin

□ ibuprofen

□ acetaminophen

□ other medication ____________

□ surgical treatment

1. Did the baby have pulmonary hypertension requiring any treatment? □Yes, □No

If Yes, complete 4a and 4b.

4a. Who made a diagnosis of pulmonary hypertension?

□ pediatric cardiologist

□ neonatologist

□ medical technician

4b. Was this diagnosis based on (Check all that apply)

□ Echocardiogram

□ Cardiac catheterization

1. Was airway endoscopy performed?
   5a. If Yes, indicate clinical findings (check all that apply):

□ No abnormality noted

□ Tracheomalacia

□ Laryngomalacia

□ Subglottic stenosis

□ Vocal cord paralysis (unilateral)

□ Vocal cord paralysis (bilateral)

□ Other, specify:______________

1. Did the baby have a tracheotomy? □Yes, □No

6a. If Yes, indicate the procedure date: ____/_____/______

Infection

1. Did he baby have any blood culture-proven Sepsis? □Yes, □No

7a. If Yes, indicate the type(s) of Sepsis:

□ Bacterial Number of distinct episodes: ___

□ Fungal Number of distinct episodes: ___

□ Viral Number of distinct episodes: ___

7b. Presumed, but not culture-proven Sepsis? □Yes, □No

If Yes, number of distinct episodes: ___

1. Did the baby have any culture-proven Meningitis?

8a. If Yes, indicate the type(s) of Meningitis:

□ Bacterial Number of distinct episodes: ___

□ Fungal Number of distinct episodes: ___

□ Viral Number of distinct episodes: ___

8b. Presumed, but not culture-proven Meningitis? □Yes, □No

If Yes, number of distinct episodes: ___

1. Did the baby have upper respiratory tract infection of confirmed viral etiology? □Yes, □No

9a. If Yes, indicate the confirmed viral etiologies (check all that apply):

□ Influenza

□ Para-influenza

□ Rhinovirus

□ Respiratory syncytial virus (RSV)

□ Other, specify ____________

1. Did the baby have any other infections? □Yes, □No

10a. If Yes, indicate the infections (check all that apply):

□ Urinary tract infection

□ Cellulitis

□ Osteomyilitis

□ Enterocolitis

□ Congenital Cytomegalovirus (CMV) infection

□ Postnatally acquired CMV infections

□ Surgical wound infection

□ Catheter associated bloodstream infection

□ Other, specify ____________

Gastro Intestinal

1. Did the baby have Necrotizing Enterocolitis (NEC) Bell stage 2 or 3? □Yes, □No

11a. If Yes, date of first medical diagnosis: ____/_____/______

11b. Were there any bowel perforations? □Yes, □No

11c. Did the baby have surgery for NEC? □Yes, □No

If Yes, indicate the surgical procedure(s) performed (check all that apply) and provide the date(s) of surgery:

□ Peritoneal drain ____/_____/______

□ Laparotomy ____/_____/______

□ Bowel resection ____/_____/______

□ Repair of Adhesion / Strictures ____/_____/______

1. Did the baby have any Isolated Bowel Perforations not considered to be associated with NEC?

□Yes, □No

12a. If Yes, date of first medical diagnosis: ___/_____/______

12b. Did the baby receive any surgery for Isolated Bowel Perforations not considered to be associated with NEC? ___/_____/______

If Yes, indicate the surgical procedure(s) performed (check all that apply) and provide the date(s) of surgery:

□ Peritoneal drain ____/_____/______

□ Laparotomy ____/_____/______

Ophthalmologic

1. Were any Retinopathy of Prematurity (ROP) examinations performed prior to discharge from the PROP study center? □Yes, □No
2. Was this baby diagnosed with ROP? □Yes, □No

If Yes, answer the following questions:

14a. What was the worst stage ever reported in any zone?

International classification Left Eye (1-5) __ Right Eye (1-5) __

Japanese classification Left Eye (1-5) __ Right Eye (1-5) __

14b. Did the baby undergo laser or cryo-surgery?

Left Eye □Yes, □No Right Eye □Yes, □No

14c. Did the baby undergo Bevacizumab (Avastin) treatment?

Left Eye □Yes, □No Right Eye □Yes, □No

14d. Did the baby undergo vitrectomy?

Left Eye □Yes, □No Right Eye □Yes, □No

Neurologic

1. Did the baby receive a subcutaneous ventricular reservoir? □Yes, □No

15a. If Yes, provide the date of first placement: ____/_____/______

1. Did the baby receive a ventriculoperitoneal shunt? □Yes, □No

16a. If Yes, provide the date of first shunt placement: ____/_____/______

Other Surgeries (excluding PDA ligation, surgery for NEC or Bowel Perforation, all surgeries for ROP, ventriculoperitoneal shunt placement, and tracheotomy)

1. Type of surgery: __________________________ Date of surgery: ____/____/______

2. Type of surgery: __________________________ Date of surgery: ____/____/______

3. Type of surgery: __________________________ Date of surgery: ____/____/______

4. Type of surgery: __________________________ Date of surgery: ____/____/______

EXTREMELY PREMATURITY AND PULMONARY OUTOCOMES PROGRAM IN SAITAMA

DISCHARGE FORM

PID: _______________ DATE: _____/______/_______

1. What was the baby’s discharge date? _____/______/_______
2. Where was the baby discharged to?

□ Home

□ Transfer to another hospital

□ Baby died at study center

□ Other, specify: __________

2a. If discharge to home, which type of home?

□ House

□ Apartment

2b. If discharge to home, what devices are required? (check all that apply):

□ Breathing and heart rate monitor

□ Oxygen therapy

□ CPAP/BIPAP/HFNC

□ Ventilator

□ Trach or breathing tube

□ Feeding Tube in nose

□ Feeding Tube in stomach

□ Other: ____________

2c. If transfer to another hospital, what was the purpose of the transfer?

□ completion of treatment

□ discharge support

2d. If baby died at study center, what was the cause of death?: _______________

1. Was this baby enrolled in NRN JAPAN database? □Yes, □No

3a. If Yes, provide study specific Participant ID ________________

1. this baby enrolled in any clinical trial? □Yes, □No

4a. If Yes, provide clinical trial name(s): ________________

1. Was this baby enrolled in any other long term follow-up studies?

5a. If Yes, provide study name(s): ________________

1. How many people normally live in your home including your baby ________

6a. How many other children under 5 years old live in this baby’s home? ________

6b. How many children between ages 5-12 years old live in this baby’s home? ________

1. Is baby exposed to dogs, cats, or other furry animals at home? □Yes, □No
2. Will your baby receive any care outside of the home in the next year?

□Yes, □No, □Unknown

1. Which one of the following three statements best describes smoking in this baby’s home?

□ Smoking is allowed anywhere in the home

□ Smoking is limited to part of the house where baby rarely goes

□ Smoking is not allowed inside the home at all

1. Which one of the following three statements best describes smoking in the car?

□ Child rarely travels by car.

□ There is no smoking inside the car

□ Smoking occurs in the car only when baby is not inside

□ Smoking is sometimes allowed in the car

□ Smoking is usually or always allowed in the car

1. Please tell us what breathing and allergy problems run in the family (Check all that apply)

| Symptoms | None/ Not applicable | Biological Siblings (any) | Biological Parents  (one or both) |
| --- | --- | --- | --- |
| a. Asthma/Recurrent  lung infections | □ | □ | □ |
| b. Allergies/Hayfever | □ | □ | □ |
| c. Eczema | □ | □ | □ |

EXTREMELY PREMATURITY AND PULMONARY OUTOCOMES PROGRAM IN SAITAMA

CONTACT FORM

ADMINISTRATIVE

PID: _______________ DATE: _____/______/_______

1. Child’s name: ______________________

1a. Child’s nickname (if any): ______________

1b. Child’s sex: □ Male □ Female

1. Child’s birth date: _____/______/_______
2. Telephone Number: ______________
3. Address: ________________________________________________________

Primary Contact Information

1. Name: __________________
2. Telephone Number: ______________
3. Cell Phone Number: ______________
4. Address: ________________________________________________________
5. Email address: ______________________________

Please provide the name and contact information of someone who is most likely to have your address in case we lose contact with you. It is helpful if this person does not reside with your child.

1. Is this the Primary Contact? □ Yes (Skip to next section) □ No (Complete contact information)
2. Name: ______________________
3. Telephone Number: ______________
4. Cell Phone Number: ______________
5. Address: ________________________________________________________
6. Email address: ______________________________
