# Supplementary material for: Extreme Prematurity and Pulmonary Outcomes Program in Saitama: Protocol for a Prospective Multicenter Cohort Study in Japan
Source: JMIR Res Protoc. 2021 Mar 5;10(3):e22948. doi: 10.2196/22948 (PMC7980118; doi:10.2196/22948)
Supplement: Multimedia Appendix 4 [file resprot_v10i3e22948_app4.docx]

EXTREME PREMATURITY AND PULMONARY OUTCOMES PROGRAM IN SAITAMA

STANDARD VISIT

PID: _______________ DATE: _____/______/_______

1. Was this interview conducted? □Yes, □No

If Yes, answer questions 1a and 1b.

1a. Visit month

□ Month 3 □ Month 6 □ Month 9 □ Month 12

1b. Date of Interview: ____/_____/______

1c. If no, indicate reason why interview was not conducted (Please select only one)

□ Unable to contact

□ Refused interview

□ Child died (Please complete Record of Death form)

□ Other, specify

1. Initials of person completing this form _________

EXTREMELY PREMATURITY AND PULMONARY OUTOCOMES PROGRAM IN SAITAMA

FOLLOW-UP INTERVIEW

PID: _______________ DATE: _____/______/_______

1. Please indicate how interview was conducted.

□ Over the phone □ In person □ In hospital

Section I: Hospitalizations and Urgent Care Visits

Since our last contact with you about your baby

1. How many times has baby been admitted to a hospital for one or more nights in a row outside of the Emergency Room?

______ times □ Don’t know

2a. How many times were because of wheezing, breathing problems or a change in his/her breathing?

______ times □ Don’t know

2b. Did any of these times require admission to an intensive care unit (NICU, PICU, or Critical Care Unit)?

□ Yes □ No □ Don’t know

2c. Were any of the admissions due to Respiratory Syncytial Virus (RSV)?

□ Yes □ No □ Don’t know

1. How many times has baby had a sick visit to a doctor’s office, clinic or Emergency Room?

______ times □ Don’t know

3a. How many times were because of wheezing, breathing problems or a change in his/her breathing?

______ times □ Don’t know

Section II: Breathing, Wheezing, and Coughing Assessment

1. Has baby’s chest sounded wheezy or whistling? □ Yes □ No □ Don’t know

If Yes, answer questions a-d.

5a. Has this occurred with colds?

□ Yes □ No □ Don’t know

5b. Has baby’s chest sounded wheezy or whistling apart from colds?

□ Yes □ No □ Don’t know

5c. How often has baby’s chest sounded wheezy during the day time?

□ Less than once per week □ 1-2 times per week □ 3-6 times per week

□ Daily, but not all the time □ Daily, all the time

5d. How often has baby’s chest sounded wheezy during the night time?

□ Less than once per week □ 1-2 times per week □ 3-6 times per week

□ Daily, but not all the time □ Daily, all the time

1. Since our last contact has baby been diagnosed with wheezing by a doctor?

□ Yes □ No □ Don’t know

Since our last contact with you about baby

1. Has baby had a cough without a cold?

□ Yes □ No □ Don’t know

If Yes, answer questions a-b.

6a. How often has baby had coughing during the day time?

□ Less than once per week □ 1-2 times per week □ 3-6 times per week

□ Daily, but not all the time □ Daily, all the time

6b. How often has baby had coughing during the night time?

□ Less than once per week □ 1-2 times per week □ 3-6 times per week

□ Daily, but not all the time □ Daily, all the time

Since our last contact with you about baby

1. How many head colds (common colds) has baby had?

□ 0 □ 1 □ 2 □ 3 □ 4 or more

Section III: Respiratory Medications

Please record all medications that the baby is currently using or has used since discharge or the last contact. Record the medication name (generic or brand name) in the Respiratory Medication worksheet.

Section IV: Home Technology Dependence

1. Since our last contact has baby used any medical equipment or any of the following in the home?

8a. Breathing and Heart Rate monitor □ Yes □ No

8b. Oxygen therapy □ Yes □ No

8c. CPAP or BIPAP □ Yes □ No

8d. Ventilator □ Yes □ No

8e. Trach or breathing tube □ Yes □ No

8f. Feeding Tube in nose □ Yes □ No

8g. Feeding Tube in stomach □ Yes □ No

8h. Other ___________________________

NOTE – The Month 3 and Month 9 questionnaires end here.

Complete the entire form at Months 6 and 12.

Section V: Nutrition

1. Since our last contact, did baby receive mother’s breast milk, either at breast, from a bottle or through a tube? □ Yes □ No □ Don’t know

9a. If Yes, for how many months did baby receive any breast milk for more than half of the feedings?

□ Less than 1 □ 1 □ 2 □ 3

Section VI: Exposure to Tobacco products and Respiratory Irritants

1. How often has the mother or primary caregiver smoked in the in the last 6 months?

□ Never □ Monthly □ Weekly □ Daily

1. How many people who live in baby’s home smoke? ______ people
2. Which one of the following three statements best describes smoking in baby’s home?

□ Smoking is allowed anywhere in the home

□ Smoking is limited to part of the house where baby rarely goes

□ Smoking is not allowed inside the home at all

Please indicate if baby is exposed to the following at HOME

1. Dogs, cats, and other furry animals □ Yes □ No

13a. If yes, how many pets indoor? ________ pets

13a. If yes, how many pets outdoor? ________ pets

1. Does your child receive regular care (at least once a week) outside the home?

□ Yes □ No

14a. If Yes, are there children that are not siblings also present at the outside care site?

□ Yes □ No

If response to Question 14 is Yes and baby attends DAY CARE, please answer questions 15,16, if not, please skip to question 17. Please indicate if baby is exposed to the following at DAY CARE

1. Smoke from cigarettes or other tobacco products? □ Yes □ No □ N/A

15a. If yes, how often is it used? □ Daily □ Weekly □ Monthly

1. Dogs, cats, and other furry animals □ Yes □ No □ N/A

15a. If yes, how many pets indoor? ________ pets

15a. If yes, how many pets outdoor? ________ pets

If response to Question 14 is Yes and baby goes to the HOME of a Babysitter or Other Regular Caregiver, please answer questions 17,18, if not, please skip to question 19.

Please indicate if <baby’s name> is exposed to the following at the HOME of BABYSITTER or Other REGULAR CAREGIVER

1. Smoke from cigarettes or other tobacco products? □ Yes □ No □ N/A

15a. If yes, how often is it used? □ Daily □ Weekly □ Monthly

1. Dogs, cats, and other furry animals □ Yes □ No □ N/A

15a. If yes, how many pets indoor? ________ pets

15a. If yes, how many pets outdoor? ________ pets

Section VII: Respiratory Treatments

1. Has baby had regular shots to prevent Respiratory Syncytial Virus (RSV)?

□ Yes □ No □ Don’t know

1. Are baby’s immunizations up to date? □ Yes □ No □ Don’t know

20a. If not, why not? □ Illness □ Refused □ Other

1. How much does baby’s health and healthcare needs disrupt your own lifestyle or other planned activities? (For example: ability to attend work or school)

□ Not at all □ Infrequently □ Half the time □ Frequently □ All of the time

Section IX: Atopy and Allergy Assessment (Completed at Month 12 ONLY)

1. Has baby had a new diagnosis of asthma or reactive airways disease diagnosed by a doctor?

□ Yes □ No □ Don’t know

1. Has baby had a runny, stuffy, or itchy nose, or watery eyes apart from a cold?

□ Yes □ No □ Don’t know

1. Has baby ever been allergic to any food?

□ Yes □ No □ Don’t know

1. Has baby been diagnosed with eczema (allergic skin rash)?

□ Yes □ No □ Don’t know

EXTREMELY PREMATURITY AND PULMONARY OUTOCOMES PROGRAM IN SAITAMA

RESPIRATORY MEDICATION WORKSHEET

PID: _______________ DATE: _____/______/_______

Please ask parent to collect all medications that the baby is currently using or has used since [discharge or the last contact] and identify them by reading the medication label or package. Record the medication name (generic or brand name) on this Respiratory Medication worksheet. There is no need to record dose, unit, frequency or route of administration.

| Respiratory Medication | Notes |
| --- | --- |
|  |  |
|  |  |
|  |  |
|  |  |
|  |  |
|  |  |
|  |  |
|  |  |
|  |  |
|  |  |
|  |  |
|  |  |
|  |  |
