# Supplementary material for: Extreme Prematurity and Pulmonary Outcomes Program in Saitama: Protocol for a Prospective Multicenter Cohort Study in Japan
Source: JMIR Res Protoc. 2021 Mar 5;10(3):e22948. doi: 10.2196/22948 (PMC7980118; doi:10.2196/22948)
Supplement: Multimedia Appendix 5 [file resprot_v10i3e22948_app5.docx]

EXTREME PREMATURITY AND PULMONARY OUTCOMES PROGRAM IN SAITAMA

BRAIN IMAGING DATA

PID: _______________ DATE: _____/______/_______

1. Was head MRI performed? □Yes, □No

1a. If Yes, indicate at which time:

□ Within 7 days +/- 1 week after baby’s birth

□ Within 30 days +/- 1 week after baby’s birth

□ Between 34 Weeks and 40 Weeks Post-Menstrual Age

1. What MRI sequence was used?

□ FLAIR (Fluid Attenuated Inversion Recovery)

□ SWI (Susceptibility Weighted Imaging)

□ WDI (Diffusion Weighted Imaging)

□ ADC (Apparent Diffusion Coefficient)

□ DTI (Diffusion Tensor Imaging)

□ MRS (MR Spectroscopy)

□ others: ____________

1. What were the results of the head MRI? Check ALL that apply.

□ Normal

□ Subependymal hemorrhage (Grade 1 hemorrhage)

□ IVH without ventricular dilation (Grade 2 hemorrhage)

□ IVH distending at least one lateral ventricle (Grade 3 hemorrhage)

□ Intraparenchymal echodense lesion (Grade 4 hemorrhage)

□ Cystic Periventricular Leucomalacia (PVL)

□ Porencephalic cyst

□ Ventriculomegaly (with or without resolving IVH)

□ Cortical atrophy

□ Cerebellar hemorrhage

□ Other, specify: ______________

1. Who interpreted MRI image?

□ Neonatologist

□ Pediatric neurologist

□ Radiologist

□ other

EXTREMELY PREMATURITY AND PULMONARY OUTOCOMES PROGRAM IN SAITAMA

RECORD OF DEATH

PID: _______________ DATE: _____/______/_______

1. What was the baby’s date of death? _____/______/_______
2. What was the baby’s primary cause of death? (Specify cause of death from the death certificate)

_________________________

1. Was an autopsy performed?

□ Yes □ No □ Unknown

3a. If Yes, what were the findings:

______________________________________________________________________________________________________________________________________________________________________

1. Was an autopsy imaging performed?

□ Yes □ No □ Unknown

3a. If Yes, what were the findings:

______________________________________________________________________________________________________________________________________________________________________

1. Please provide a short description of the infant’s underlying condition prior to the events leading to the infant’s death and the circumstances leading to the infant’s death.

____________________________________________________________________________________________________________________________________________________________________________________________________________________________________________________________________________________________________________________________________________

1. Do you think that a cardiopulmonary disorder contributed to the death of this infant?

□ Yes □ No □ Cannot make this determination

EXTREMELY PREMATURITY AND PULMONARY OUTOCOMES PROGRAM IN SAITAMA

STUDY STATUS

PID: _______________ DATE: _____/______/_______

NOTE: This form must be completed when the infant's participation in the study ends early.

1. Date of last contact? ____/____/________
2. Indicate the primary reason participation stopped:

□ Unable to contact parents/caregivers

□ Parents/Caregivers refuse further participation

□ Other, specify _____________________

EXTREMELY PREMATURITY AND PULMONARY OUTOCOMES PROGRAM IN SAITAMA

RELOCATION CONTACT INFORMATION

PID: _______________ DATE: _____/______/_______

1. Date of relocation: _____/______/_______
2. New Clinical Center: ___________________________

□ EPOPS site □ non- EPOPS site

1. New Address:

___________________________________________________________________

1. New Telephone Number: ___________________________
