# Supplementary material for: Extreme Prematurity and Pulmonary Outcomes Program in Saitama: Protocol for a Prospective Multicenter Cohort Study in Japan
Source: JMIR Res Protoc. 2021 Mar 5;10(3):e22948. doi: 10.2196/22948 (PMC7980118; doi:10.2196/22948)
Supplement: Multimedia Appendix 6 [file resprot_v10i3e22948_app6.docx]

**Multimedia Appendix 6. Oxygen requirement challenge test**

The infants are monitored in quiet sleep for at least 30 min after feeding. If oxygen saturations are maintained greater than or equal to 90% for 15 min on the prescribed nasal cannula support, the FiO2 is weaned to 0.21 in reductions of 0.2 at 5-min intervals. Subsequently, the flow is reduced in 1 liter/min reductions at 10-min intervals until it is less than 1.5 liter/min, followed by 50% reductions to a minimum of 0.125 liter/min. After a 10-min observational period, the nasal cannula is removed and the infant is monitored in room air for 1 h. Failure at any point during this challenge is defined as: (1) SpO2 less than 90% for 5 continuous min; (2) SpO2 less than 80% for 15 sec; or (3) apnea for more than 20 sec. If the duration of apnea exceeds 20 sec and the rate of bradycardia is less than 80 beats/min for more than 10 sec, they are recorded as adverse events. The infants are then returned to the original support at the end of this challenge.
