# Supplementary material for: Extreme Prematurity and Pulmonary Outcomes Program in Saitama: Protocol for a Prospective Multicenter Cohort Study in Japan
Source: JMIR Res Protoc. 2021 Mar 5;10(3):e22948. doi: 10.2196/22948 (PMC7980118; doi:10.2196/22948)
Supplement: Multimedia Appendix 7 [file resprot_v10i3e22948_app7.docx]

**Multimedia Appendix 7. Institutional review board protocols at individual Extreme Prematurity and Pulmonary Outcomes Program in Saitama sites.**

1. Saitama Medical Center, Saitama Medical University – 2168
2. Saitama City Hospital – A3148
3. Saitama Children's Medical Center –2019-05-028
4. Saitama Medical Center, Jichi Medical University – under review
5. Saitama Medical University Hospital – 19114.01
6. Kawaguchi Municipal Medical Center – March the 3rd, 2020 approved
